# Accuracy of Immunodiagnostic Tests for Active Tuberculosis Using Single and Combined Results: A Multicenter TBNET-Study

**DOI:** 10.1371/journal.pone.0003417

**Published:** 2008-10-15

**Authors:** Delia Goletti, Carrara Stefania, Ornella Butera, Massimo Amicosante, Martin Ernst, Ilaria Sauzullo, Vincenzo Vullo, Daniela Cirillo, Emanuele Borroni, Roumiana Markova, Roumiana Drenska, José Dominguez, Irene Latorre, Claudio Angeletti, Assunta Navarra, Nicola Petrosillo, Francesco Nicola Lauria, Giuseppe Ippolito, Giovanni Battista Migliori, Christoph Lange, Enrico Girardi

**Affiliations:** 1 Translational Research Unit, Department of Epidemiology and Preclinical Research, National Institute for Infectious Diseases (INMI) L. Spallanzani, IRCCS, Rome, Italy; 2 Department of Internal Medicine, University “Tor Vergata”, Rome, Italy; 3 Division of Clinical Infectious Diseases, Research Center Borstel, Borstel, Germany; 4 Infectious and Tropical Diseases Department, “La Sapienza” University, Rome, Italy; 5 Emerging Bacterial Pathogens Unit, “San Raffaele” Scientific Institute, Milan, Italy; 6 Department of Immunology and Allergology, National Center for Infectious and Parassitic Diseases, Sofia, Bulgaria; 7 Fundació Institut d'Investigació en Ciències de la Salut Germans Trias i Pujol, CIBER Enfermedades Respiratorias, Universitat Autònoma de Barcelona, Barcelona, Spain; 8 Clinical Epidemiology, Department of Epidemiology and Preclinical Research, INMI “L.Spallanzani”, Rome, Italy; 9 Clinical Department, INMI “L.Spallanzani”, Rome, Italy; 10 WHO Collaborating Centre for Tuberculosis and Lung Diseases, S. Maugeri Foundation, IRCCS, Tradate, Italy; McGill University, Canada

## Abstract

**Background:**

The clinical application of IFN-γ release assays (IGRAs) has recently improved the diagnosis of latent tuberculosis infection. In a multicenter study of the Tuberculosis Network European Trialsgroup (TBNET) we aimed to ascertain in routine clinical practice the accuracy of a novel assay using selected peptides encoded in the mycobacterial genomic region of difference (RD) 1 for the diagnosis of active tuberculosis in comparison with tuberculin skin test (TST), QuantiFERON-TB GOLD In-Tube (Cellestis Ltd., Carnegie, Australia) and T-SPOT.*TB* (Oxfordimmunotec, Abingdon, UK).

**Principal Findings:**

425 individuals from 6 different European centres were prospectively enrolled. We found that sensitivity of the novel test, TST, QuantiFERON-TB GOLD In-Tube and T-SPOT.*TB* was respectively 73.1%, 85.3%, 78.1%, and 85.2%; specificity was respectively 70.6%, 48.0%, 61.9% and 44.3%; positive likelihood ratios were respectively 2.48, 1.64, 2.05, and 1.53; negative likelihood ratios were respectively 0.38, 0.31, 0.35, 0.33. Sensitivity of TST combined with the novel test, QuantiFERON-TB GOLD In-Tube and T-SPOT.*TB* increased up to 92.4%, 97.7% and 97.1%, respectively. The likelihood ratios of combined negative results of TST with, respectively, the novel test, QuantiFERON-TB GOLD In-Tube and T-SPOT.*TB* were 0.19, 0.07 and 0.10.

**Conclusions:**

The assay based on RD1 selected peptides has similar accuracy for active tuberculosis compared with TST and commercial IGRAs. Then, independently of the spectrum of antigens used in the assays to elicit mycobacterial specific immune responses, the novel test, IGRAs, and the TST do not allow an accurate identification of active tuberculosis in clinical practice. However, the combined use of the novel assay or commercial IGRAs with TST may allow exclusion of tuberculosis.

## Introduction

Tuberculosis control is based on the consequent use of preventive chemotherapy in individuals with latent tuberculosis infection (LTBI) who are at risk of developing active disease and on the rapid diagnosis and effective treatment of infectious cases [1]–[3]. While the identification of patients with active tuberculosis can rapidly be established by the detection of alcohol acid fast bacilli (AFB) on sputum smears, early diagnosis of infectious cases by sputum microscopy is only possible in approximately 50% of cases [4]. The sub-optimal performances of existing diagnostic tools [4], in terms of both speed and sensitivity, delayed diagnosis and, consequently, treatment of active tuberculosis.

The recent introduction of T-cell-based interferon (IFN)-γ release assays (IGRAs), using antigens belonging to *M. tuberculosis* region of difference (RD) 1 (including early secreted antigenic target [ESAT]-6 and culture filtrate protein 10 [CFP]-10) represents a significant step towards improved LTBI diagnosis [5]–[9]. There is growing evidence that in low incidence settings both the commercial IGRAs currently available, the Quantiferon-GOLD In-tube assay (Cellestis Ltd., Carnegie, Australia) and the T-SPOT.*TB* assay (Oxfordimmunotec, Abingdon, UK) are less affected by bacillus Calmette-Guerin (BCG) vaccination than the tuberculin skin test (TST) and that they are more specific and correlate better with exposure to an infected index case [10]–[13]. Although these commercial assays provide an accurate diagnosis of *M. tuberculosis* infection and detect active tuberculosis disease, they cannot discriminate between active tuberculosis and LTBI. Thus, further clinical workup is required to rule out active tuberculosis after a positive response to these tests.

Recently the design of a novel *in vitro* immune diagnostic enzyme-linked immunospot (ELISPOT) and whole blood ELISA (WBE) for IFN-γ using multiepitopic peptides that are selected by computational analysis from CFP-10 and ESAT-6 as stimulating antigens has been reported [14]. It has been shown that the response to RD1 selected peptides can be detected in subjects with ongoing *M. tuberculosis* replication, such as during active tuberculosis and/or recent infection [15]–[17]. This response is mediated by CD4^+^ T effector cells, shown to undergo clonal expansion during *M. tuberculosis* replication, followed by a contraction phase after efficacious therapy culminating in the generation of CD4^+^ memory T-cells [18], [19]. These studies were conducted at one center in Italy, a country with a low tuberculosis incidence of <10/100.000 [20]. The aims of this multicenter study were: i) to evaluate whether this assay based on RD1 selected peptides may help in providing evidence of active tuberculosis; ii) to compare the response to this novel assay with TST and the commercially available RD1 tests, individually and in combination for the diagnostic work-up of active tuberculosis [21].

## Materials and Methods

### Study design

Following obtaining of informed consent, patients with a clinical suspicion of tuberculosis (abnormal chest radiograph suggestive of tuberculosis and/or other signs and symptoms such as persistent cough, haemoptysis, weight loss, fever) were prospectively recruited at participating centers of the Tuberculosis Network European Trialsgroup (TBNET): Bulgaria (Department of Immunology and Allergology, National Center for Infectious and Parassitic Diseases, Sofia), Germany (Medical Clinic, Research Centre Borstel, Borstel), Italy (INMI and University La Sapienza, Rome; Scientific Institute San Raffaele, Milan) and Spain (Hospital Universitari Germans Trias i Pujol; Barcelona) between November 2005 and March 2008.

Patients underwent clinical and microbiological examinations including chest radiographs to confirm or exclude the diagnosis of tuberculosis. Briefly, 3 sequential respiratory expectorated or 2 induced sputum smears over the first 7 days following clinical evaluation were collected. AFB smear and culture (on both, Lowenstein-Jensen and Bactec MGIT, BD Biosciences Division, Sparks, Maryland, USA) were performed on each specimen. Additionally *M. tuberculosis*-specific RNA amplification was performed on specimens from patients with a high likelihood for tuberculosis in which examinations for AFB were negative [(Gen-Probe® Amplified™ Mycobacterium Tuberculosis Direct (MTD) Test, San Diego, CA, USA)]. TSTs were administered by the Mantoux method with bioequivalent 5 Tuberculin Units (Biocine, Chiron, Siena, Italy) or 2 Tuberculin Units (RT23, Statens Serum Institute, Copenhagen, Denmark) [22], [23] or 5 Units of PPD Tuberculin Mammalian (BulBio-NCIPD, Sofia, Bulgaria). Indurations were measured 48–72 hours following tuberculin administration by the ballpoint technique. Individuals with an induration ≥10 mm [24] or in Bulgaria ≥15 mm [25]–[26] for those with past BCG vaccination were classified as TST-positive [22].

For extra-pulmonary tuberculosis, *M. tuberculosis*-specific RNA amplification (MTD Test) and/or nucleic acid amplification test (NAT) for *M. tuberculosis*-specific DNA based on a commercial test (BD ProbeTec ET system; BD Diagnostic Systems, Sparks, MD) or based on a homemade version developed from the literature [27] was performed on biopsy specimens and/or biological fluids; moreover histology and AFB staining were performed on biopsies.

Enrolled patients were classified as “confirmed tuberculosis” if the diagnosis was based: i) in those with pulmonary tuberculosis by a positive culture for *M. tuberculosis*; ii) in those with extra-pulmonary tuberculosis by a) positive *M. tuberculosis*-specific RNA amplification and/or *M. tuberculosis*-specific NAT from biological specimens *or* b) by histological pathological finding consistent with tuberculosis and presence of AFB in a tissue sample *or* c) by positive culture for *M. tuberculosis* in clinical samples (pleural fluid and abscesses). Conversely, patients were classified as “clinical tuberculosis” if the diagnosis was based on clinical and radiologic criteria (having excluded other disease) including appropriate response to anti-tuberculosis therapy.

We defined patients without tuberculosis as those admitted with a suspicion of active tuberculosis, who subsequently showed negative sputum for AFB smear and culture for *M. tuberculosis* with either a resolution of clinical symptoms and radiographic abnormalities after an antibiotic therapy not involving *M. tuberculosis* active drugs, or presenting a confirmed alternative diagnosis (e.g.: lung cancer).

Following admission, a 10–20 ml (depending on the center) heparin venous blood sample was drawn from all enrolled individuals. ELISPOT or WBE based on RD1 selected peptides was performed. In a subgroup of patients the test was done in parallel with the commercially available immune assays for tuberculosis. Clinicians were blinded to the results of *in vitro* assays and laboratory personnel were blinded to the status of the patient. The study was approved by the ethics committee at all the institutions in which the study was performed.

### RD1 selected peptides and stimuli used for cell cultures

The selection of Human Leukocytes Antigens (HLA)-class II restricted epitopes of ESAT-6 and CFP-10 *M. tuberculosis* proteins was performed by quantitative implemented HLA peptide-binding motifs analysis as previously described for ESAT-6 [14], [15]. Peptides were synthesized as free amino acid termini using Fmoc chemistry (ABI, Bergamo, Italy). Lyophilized peptides were diluted in DMSO at stock concentrations of 10 mg/mL for each peptide and stored at −80°C. RD1 selected peptides were used as follows: a pool of the two ESAT-6 peptides (at 10 µg/mL each), a pool of the three CFP-10 peptides (at 2 µg/mL each). DMSO was used as negative control at 10 µg/mL. As positive control we used Phytohemagglutinin (PHA) (Sigma, St Louis, MO, USA) at 5 µg/mL. RD1 selected peptides from the same batch were provided to the all centers with a detailed protocol. Four out of the 5 external centers received personal training from INMIs' laboratory personnel for at least 2-days. Inter-site communication was present all over the performance of the study to solve any potential problem.

#### ELISPOT

2.5×10^5^ peripheral blood mononuclear cells (PBMC) were separated, washed twice and plated in the T-SPOT.*TB* plates stimulated with or without RD1 selected peptides and PHA, as described above and previously [14], [15]. Cell cultures were incubated overnight at 37°C, with 5% CO2. On the next morning, the cells were washed off, and the ELISPOT was developed following the manufacturer's instructions (Oxford Immunotec, UK). Spots were then counted by an automated ELISA-Spot assay video analysis system (AELVIS, Hannover, Germany). Evaluated spots had a size >15 U (1 U = 50 µm2). Indeterminate results were defined by values in the PHA-stimulated samples below 34 spot-forming cells per million PBMC. The RD1 selected peptide responses were scored as positive if above 34 spot-forming cells/million PBMC. This cutoff value was determined by constructing a receiver operator characteristic (ROC) curve by means of LABROC-1 software. To obtain the absolute value, the number of spot-forming cells in the negative controls was subtracted from the number of spot-forming cells in the stimulated cultures. Clinicians were blinded to the laboratory test results and laboratory personnel were blinded to the status of the patients.

#### WBE

Briefly, aliquots of 0.5 ml per well of heparinized blood were seeded in a 48-well plate and stimulated with or without RD1 selected peptides and PHA, as described above. Samples were then incubated for 24 hours at 37°C in presence of 5% CO_2_ when an amount of 100 µl of plasma was harvested. IFN-γ levels in culture supernatants were assessed by a commercially available kit (QuantiFERON-CMI kit, Cellestis). For the results scoring, a cut-off value of 0.7 IU/mL was chosen for all stimuli by constructing a ROC curve. Indeterminate results were defined by values in the PHA-stimulated samples below 0.7 IU/mL.

### Commercially available assays

T-SPOT.*TB* and QuantiFERON-TB GOLD In-Tube assays were performed and their results were scored as indicated by the manufacturers.

#### Statistical Analysis

The tests performance was evaluated by using categories of confirmed tuberculosis, clinical tuberculosis, and no active tuberculosis. Cases with indeterminate responses to *in vitro* assays were not included in the analysis. Sensitivity, specificity and likelihood ratios with their 95% Confidence Interval (CI), were computed for each test overall and according to the diagnostic categories and tuberculosis localization. Proportions were compared by using Fisher exact test and, for paired data, McNemar chi-square test. Sensitivities of 2 tests used in combination were obtained assuming that a positive result is given by a positive response to at least one assay.

Furthermore the accuracy of two tests used in combination was analyzed by computing the likelihood ratios, together with the distribution of subjects with and without active tuberculosis, according to the responses to the tests. Sensitivities and specificities of diagnostic tests were compared by using a logistic regression model with robust standard errors to account for the correlation between observations. Two-tailed *P* values are reported.

## Results

**Figure 1:**
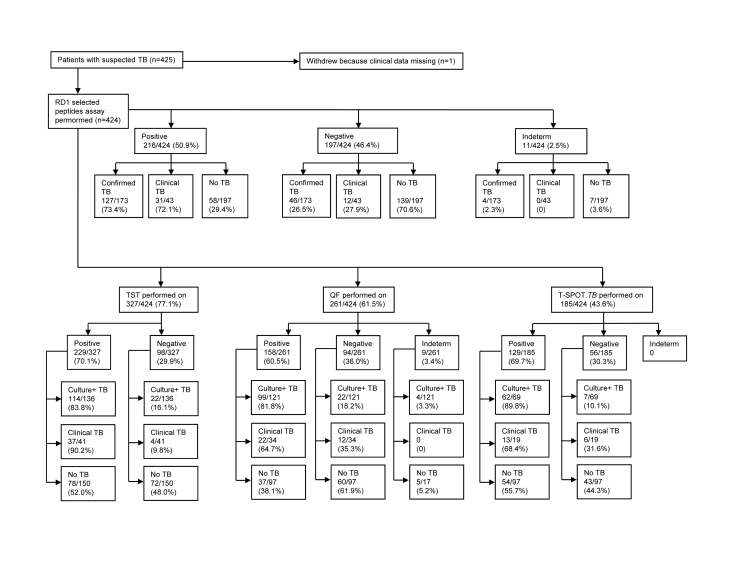
Study flow diagram. Abbreviations: TB: tuberculosis; RD: Region of Difference; Indeterm: indeterminate; TST: tuberculin skin test; QF: QuantiFERON-TB GOLD In-Tube.

We consecutively enrolled 425 consenting adult patients from 6 different centres in Europe. Complete data were unavailable from 1 patient. Results from 11 (2.5%) subjects were found to be indeterminate by *in vitro* assays based on RD1 selected peptides and/or QuantiFERON-TB GOLD In-Tube (Figure 1). Among them, 4 had active tuberculosis and 7 were without active tuberculosis. These patients were similar to those without indeterminate results in terms of age, sex, ethnicity, immune suppressive therapy intake and presence of comorbidity conditions (data not shown).

Hereafter, the data analysis was performed only in the 413 samples with valid *in vitro* test results. Demographic characteristics of these subjects are shown in Table 1. Among them we classified 173 patients (41.9%) as having confirmed tuberculosis and 43 (10.4%) as having clinical tuberculosis. We excluded active tuberculosis in 197 patients (47.7%). Based on localization site, 146 (67.6%) were classified as having pulmonary tuberculosis, 56 (25.9%) extrapulmonary tuberculosis and 14 (6.5%) had both pulmonary and extrapulmonary localization (Table 2).

**Table 1:** Demographic and clinical characteristics of the subjects enrolled in the study.

	Confirmed TB	Clinical TB	No Active TB	Total
	N. 173 (%)	N. 43 (%)	N. 197 (%)	N. 413 (%)
**Age years (median)**	34	36	48	40
**Gender**				
**Female**	69 (39.9)	15 (34.9)	67 (34.0)	151 (36.6)
**Male**	104 (60.1)	28 (65.1)	130 (66.0)	262 (63.4)
**BCG**				
**Yes**	105 (60.6)	7 (16.2)	54 (27.4)	166 (46.0)
**No**	54 (31.2)	34 (79.0)	107 (54.3)	195 (54.0)
**Unknown**	14 (8.0)	2 (0.04)	36 (18.2)	52 (12.5)
**Origin**				
**Africa**	29 (16.8)	6 (14.0)	11 (5.6)	46 (11.1)
**Asia**	17 (9.8)	10 (23.3)	9 (4.6)	36 8.7)
**Eastern Europe**	66 (38.2	5 (11.6)	31 (15.7)	102 (24.7)
**South America**	17 (9.8)	0	10 (5.1)	27 (6.5)
**Western Europe**	44 (25.4)	22 (51.2)	136 (69.0)	202 (48.9)
**Past TB**				
**Yes**	0	0	27 (13.7)	27 (6.5)
**No**	173 (100)	43 (100)	170 (86.3)	386 (93.5)
**Immune suppressive therapy**				
**Yes**	4 (2.3)	2 (4.7)	4 (2.0)	10 (2.4)
**No**	169 (97.7)	41 (95.3)	193 (98.0)	403 (97.6)
**HIV status**				
**Yes**	3 (1.7)	0	4 (2.0)	7 (1.7)
**No**	155 (89.5)	43 (100)	167 (84.7)	365 (88.3)
**Unknown**	15 (8.6)	0	26 (13.2)	41 (9.9)

**Abbreviations:**

TB: tuberculosis; BCG: Bacillus Calmette and Guerin; HIV: Human Immunodeficiency Virus.

**Table 2:** Accuracy for the diagnosis of active tuberculosis.

Sensitivity*		RD1 selected peptides test	TST	QuantiFERON-TB GOLD In-Tube	T-SPOT.*TB*
		Positive over total (%) [CI]			
**According to diagnostic criteria**	**Confirmed TB**	127/173 (73.4)	114/136 (83.8)	99/121 (81.8)	62/69 (89.9)
		[66.2–79.8]	[76.5–89.6]	[73.8–88.2]	[80.2–95.8]
	**Clinical TB**	31/43 (72.1)	37/41 (90.2)	22/34 (64.7)	13/19 (68.4)
		[56.3–84.7]	[76.9–97.3]	[46.5–80.3]	[43.4–87.4]
**According to TB localization**	**Pulmonary**	109/146 (74.7)	100/115 (87.0)	88/107 (82.2)	49/56 (87.5)
		[66.8–81.5]	[79.4–92.5]	[73.7–89.0]	[75.9–94.8]
	**Extra-pulmonary**	40/56 (71.4)	41/47 (89.4)	26/39 (66.7)	17/23 (73.9)
		[57.8–82.7]	[76.9–96.5]	[49.8–80.9]	[51.6–89.8]
	**Pulmonary and extra-pulmonary**	9/14 (64.3)	9/15 (60.0)	7/9 (77.8)	9/9 (100)
		[35.1–87.2]	[32.3–83.7]	[40.0–97.2]	[71.7–100.0]
**Total**		158/216 (73.1)	151/177 (85.3)	121/155 (78.1)	75/88 (85.2)
		[66.7–78.9]	[79.2–90.2]	[70.7–84.3]	[76.1–91.9]
**Specificity** **					
		139/197 (70.6)	72/150 (48.0)	60/97 (61.9)	43/97 (44.3)
		[63.7–76.8]	[39.8–56.3]	[51.4–71.5]	[34.2–54.8]
**Positive likelihood ratio** ***					
		2.48	1.64	2.05	1.53
		[1.97–3.1]	[1.39–1.94]	[1.57–2.67]	[1.26–1.87]
**Negative likelihood ratio** ***					
		0.38	0.31	0.35	0.33
		[0.30–0.48]	[0.21–0.45]	[0.25–0.50]	[0.19–0.58]

* evaluated on the total number of positive results over the total number of patients with active tuberculosis disease.

** evaluated on the total number of negative results over the total number of patients without active tuberculosis disease.

*** evaluated on the total number of tuberculosis cases (confirmed and clinical tuberculosis).

**Abbreviations:**

TB: tuberculosis; RD: region of difference; TST: tuberculin skin test; CI: confidence interval.

### Response to RD1 selected peptides assay and comparison with the other tests

#### WBE and ELISPOT readouts significantly correlate for the detection of the responses to RD1 selected peptides

Evaluation of the response to RD1 selected peptides was performed by 2 different readouts, the ELISPOT and the WBE that we previously demonstrated to significantly correlate with each other [15]. Also in this study, 138 samples were run in parallel with a significant correlation (percentage of agreement: 80.4%; p = 0.0001). Moreover no differences were found in terms of detection of positive results in those with active tuberculosis among the patients from the different centers (p>0.5). Given the concordance of the results, the data were pooled together and analyzed as a whole.

### Response to immunological tests for tuberculosis: assay based on RD1 selected peptides, TST, commercial IGRAs

For confirmed and clinical tuberculosis cases, diagnostic test sensitivities were 73.1% (95% CI, 66.7–78.9%) with RD1 selected peptides test, 85.3% (95%CI, 79.2–90.2%) with TST, 78.1% (95% CI, 70.7–84.3%) with QuantiFERON-TB GOLD In-Tube, and 85.2% (CI, 76.1–91.9%) with T-SPOT.*TB* (Table 2).

To investigate whether inclusion of patients with clinical tuberculosis in the analysis affected performance estimates, we re-estimated sensitivity by using only confirmed cases. Sensitivity remained stable and was 73.4% (CI, 66.2–79.8) with RD1 selected peptides test, 83.8% (CI, 76.5–89.6) with TST, 81.8% (CI, 73.8–88.2%) with QuantiFERON-TB GOLD In-Tube, and 89.9% (CI,80.2–95.8%) with T-SPOT.*TB* (Table 2). No differences were found between the results obtained considering confirmed tuberculosis vs. clinical tuberculosis cases with the exception of T-SPOT.*TB* for which a higher proportion of positive results was observed for confirmed tuberculosis (62/69) vs. clinical tuberculosis (13/19, p = 0.03). Results of immune responses were therefore evaluated for patients with confirmed and clinical tuberculosis pooled together, unless differently specified, and were defined as patients with active tuberculosis.

Among patients with active tuberculosis, the RD1 selected peptides assay was less sensitive than TST (in the 170 patients with results from both tests) (p = 0.004) and T-SPOT.*TB* (in the 88 patients with results from both tests) (p = 0.008), but not the QuantiFERON-TB GOLD In-Tube (in the 154 patients with results from both tests) (p = 0.16).

We also evaluated the sensitivities of the different tests based on tuberculosis localization. In Table 2, sensitivities for pulmonary, extra-pulmonary and disseminated tuberculosis (pulmonary and extra-pulmonary tuberculosis) are shown. Considering each test per se, no significant difference in proportion of positive results was observed in patients with active tuberculosis according to tuberculosis localization [with the exception of TST for which the highest proportion of positive results was recorded among patients with pulmonary tuberculosis (p = 0.027)].

Of the 132 patients with culture confirmed pulmonary tuberculosis, 28 had a negative sputum smear. Among these patients, sensitivity results were 64.3% (18/28; CI, 44.1–81.4) for RD1 selected peptides test, 84.6% (22/26; CI, 65.1–95.6) for TST, 88.0% (22/25; CI, 68.8–97.5) for QuantiFERON-TB GOLD In-Tube, and 83.3% (5/6; CI, 35.9–99.6) for T-SPOT.*TB*. Compared to RD1 selected peptides test, the sensitivity for active tuberculosis was significantly higher only for QuantiFERON-TB GOLD In-Tube (p = 0.037). *M. tuberculosis*-specific RNA amplification was performed in 22 of these 27 subjects (8 sputa and 16 broncholavage) and resulted positive in 75% of sputa (6/8; CI, 34.9–96.8) and in 93.8% of broncholavages (15/16; CI, 69.8–99.8) with an overall sensitivity of 86.4% (19/22; CI, 65.1–97.1). Among the 118 patients with smear positive culture confirmed pulmonary tuberculosis the sensitivity results were 77.1% (91/118; CI, 68.5–84.3) by RD1 selected peptides test, 87.6% (78/89; CI, 79.0–93.7) by TST, 80.5% (66/82; CI, 70.3–88.4) by QuantiFERON-TB GOLD In-Tube, and 88.0% (44/50; CI, 75.7–95.5) by T-SPOT.*TB*. No statistical difference was found between the single tests' results obtained in those smear positive vs those smear negative.

Specificity for active tuberculosis was 70.6% (CI, 63.7–76.8%) with RD1 selected peptides test, 48.0% (CI, 39.8–56.3%) with TST, 61.9% (CI, 51.4–71.5%) with QuantiFERON-TB GOLD In-Tube, and 44.3% (CI, 34.2–54.8%) with T-SPOT.*TB* (Table 2). The specificity for active tuberculosis was significantly higher for the assay based on RD1 selected peptides compared with TST (p<0.001) and T-SPOT.*TB* (p<0.001).

Then we assessed whether the pair-wise combination of the tests could lead to a better evaluation of active tuberculosis diagnosis and we calculated the probabilities for the potential outcomes (double positive, double negative and discordant results) given the disease status.

The probability of a positive result of at least one of the two tests considered was higher than sensitivities of each test (Table 3). In particular the assay based on RD1 selected peptides combined with TST led to a sensitivity of 92.4% (CI, 87.3–96.0%), with QuantiFERON-TB GOLD In-Tube of 85.7% (CI, 79.2–90.8%), and with T-SPOT.*TB* of 88.6% (CI, 80.1–94.4%) (Table 3). To note that the highest sensitivities were obtained by using the combination of TST with either QuantiFERON-TB GOLD In-Tube [sensitivity of 97.7% (CI, 93.0–99.5%)], or T-SPOT.*TB* [sensitivity *of* 97.1% (CI, 89.9–94.4%) (Table 3)]. Then we estimated the likelihood ratios for the combination of the tests. Positive results from both tests provided likelihood ratios of 2.92 for the RD1 selected peptides test combined with TST [CI, 2.15–3.98], 3.21 with QuantiFERON-TB GOLD In-tube, [CI, 2.11–4.89], and 2.20 with T-SPOT.*TB* [CI, 1.59–3.07], (Table 4).

**Table 3:** Estimates of sensitivities for the combination of the diagnostic tests studied.

	Combined tests % [CI]		
	TST	QuantiFERON-TB GOLD In-Tube	T-SPOT.*TB*
**RD1 selected peptides test**	92.4 [87.3–96.0]	85.7 [79.2–90.8]	88.6 [80.1–94.4]
**TST**	-	97.7 [93.0–99.5]	97.1 [89.9–99.6]
**QuantiFERON-TB GOLD In-Tube**	-	-	87.0 [76.7–93.9]

**Abbreviations:**

RD: region of difference; TST: tuberculin skin test; CI: confidence interval.

**Table 4:** Estimates of likelihood ratios for the combination of the diagnostic tests studied according to the disease status.

Combined tests	Subjects with Active TB n (%)	Subjects without Active TB n (%)	Combined likelihood ratio [CI]
**RD1 test pos/TST pos**	116 (68.1)	35 (23.3)	2.92 [2.15–3.98]
**RD1 test neg/TST neg**	13 (7.7)	60 (40.0)	0.19 [0.11–0.33]
**RD1 test pos/TST neg**	11 (6.5)	12 (8.00)	0.81 [0.37–1.78]
**RD1 test neg/TST pos**	30 (17.7)	43 (28.7)	0.62 [0.41–0.93]
**Total**	170 (100.0)	150 (100.0)	
**RD1 test pos/QuantiFERON pos**	99 (64.3)	19 (20.0)	3.21 [2.11–4.89]
**RD1 test neg/ QuantiFERON neg**	22 (14.3)	55 (57.8)	0.25 [0.16–0.38]
**RD1 test pos/ QuantiFERON neg**	12 (7.8)	3 (3.2)	2.47 [0.71–8.52]
**RD1 test neg/ QuantiFERON pos**	21 (13.6)	18 (19.0)	0.72 [0.40–1.28]
**Total**	154 (100.0)	95 (100.0)	
**RD1 test pos/T-SPOT.** ***TB*** ** pos**	60 (68.1)	30 (30.9)	2.20 [1.59–3.07]
**RD1 test neg/T-SPOT.** ***TB*** ** neg**	10 (11.4)	41 (42.3)	0.27 [0.14–0.50]
**RD1 test pos/T-SPOT.** ***TB*** ** neg**	3 (3.4)	2 (2.1)	1.65 [0.28–9.67]
**RD1 test neg/T-SPOT.** ***TB*** ** pos**	15 (17.1)	24 (24.7)	0.69 [0.39–1.23]
**Total**	88 (100.0)	97 (100.0)	
**QuantiFERON pos/TST pos**	81 (65.8)	23 (30.7)	2.15 [1.49–3.09]
**QuantiFERON neg/TST neg**	3 (2.4)	27 (36.0)	0.07 [0.02–0.22]
**QuantiFERON pos/TST neg**	19 (15.5)	9 (12.0)	1.29 [0.61–2.70]
**QuantiFERON neg/TST pos**	20 (16.3)	16 (21.3)	0.76 [0.42–1.38]
**Total**	123 (100.0)	75 (100.0)	
**QuantiFERON pos/T-SPOT.** ***TB*** ** pos**	50 (72.5)	20 (41.7)	1.74 [1.21–2.51]
**QuantiFERON neg/T-SPOT.** ***TB*** ** neg**	9 (13.0)	24 (50.0)	0.26 [0.13–0.51]
**QuantiFERON pos/T-SPOT.** ***TB*** ** neg**	2 (2.9)	0 (0.0)	NA
**QuantiFERON neg/T-SPOT.** ***TB*** ** pos**	8 (11.6)	4 (8.3)	1.39 [0.44–4.36]
**Total**	69 (100.0)	48 (100.0)	
**TST pos/T-SPOT.** ***TB*** ** pos**	50 (72.4)	29 (36.7)	1.97 [1.43–2.73]
**TST neg/T-SPOT.** ***TB*** ** neg**	2 (2.9)	23 (29.1)	0.10 [0.02–0.41]
**TST pos/T-SPOT.** ***TB*** ** neg**	5 (7.3)	10 (12.7)	0.57 [0.21–1.59]
**TST neg/T-SPOT.** ***TB*** ** pos**	12 (17.4)	17 (21.5)	0.81 [0.42–1.57]
**Total**	69 (100.0)	79 (100.0)	

**Abbreviations:**

TB: tuberculosis; RD: region of difference; RD1 test: test based on the RD1 selected peptides; TST: tuberculin skin test; CI: confidence interval; pos: positive; neg: negative; QuantiFERON: QuantiFERON-TB GOLD In-Tube.

Negative results on combined tests were associated with lower negative likelihood ratios compared to that obtained by single assay especially when a blood test was associated with TST. In particular the negative likelihood ratio of the combination of RD1 selected peptides test with TST was 0.19 (CI, 0.11–0.33), with QuantiFERON-TB GOLD In-Tube was 0.25 (CI, 0.16–0.38) and with T-SPOT.*TB* was 0.27 (CI, 0.14–0.50) (Table 4). To note that better negative likelihood ratios were obtained by the combination of commercial tests with TST (Table 4).

## Discussion

We present the results of a prospective multicenter trial of the TBNET that was designed to investigate the performance of a novel blood test based on RD1 selected peptides for the immunodiagnosis of active tuberculosis.

The novel assay had a higher specificity for active tuberculosis than the TST and commercial IGRAs, but it had a lower sensitivity. Although the novel assay had a higher likelihood ratio, none of the tests evaluated was accurate enough to discriminate patients with active tuberculosis from those without, probably because of the high levels of LTBI in the population studied. Combined use of TST with either the RD1 selected peptides test or with the other commercial IGRAs improved the diagnostic accuracy for active disease, especially when considering the combination of negative results, contributing to rapid exclusion of tuberculosis. However, *M. tuberculosis* culture remains the diagnostic gold standard for active tuberculosis and is required for identifying drug resistance. Consequently, active tuberculosis should not be ruled out in a high-risk individual without a thorough microbiological work-up for tuberculosis disease.

The specificity of the assay based on RD1 selected peptides was lower in this multicenter trial compared with earlier, smaller studies of more limited patient groups [15], [16]. Nevertheless, also in the present study the test based on RD1 selected peptides maintains the higher specificity compared to commercial IGRAs and TST which is not unexpected. In fact, the commercial IGRAs and TST use a greater variety of epitopes to elicit *M. tuberculosis* immune responses by effector memory T-cells [28]–[29] being TST a crude preparation of several mycobacterial antigens, and commercially IGRAs based on pools of overlapping peptides spanning the whole length of CFP-10 and ESAT-6 proteins [5]. Conversely the selective approach of the design of the test based on RD1 selected peptides reduces false positive test results at the cost of a loss of diagnostic sensitivity [14]–[16]. Which would be more acceptable between false positive test results that may lead to overtreatment or false negative test results that potentially lead to missing of cases with active tuberculosis to be treated, is a matter of debate and is largely dependent upon the prevalence of *M. tuberculosis* infection and the pre-test probability of tuberculosis in a community.

Specificity of commercial IGRAs is considerably lower in the present study compared to what was reported in a recent updated meta-analysis [30]. This may be due to the fact that this report involve the enrollment of patients with a suspicion of active tuberculosis that could by affected also by LTBI, while the literature reported in the meta-analyses [30] enclosed low-risk subjects with no known tuberculosis exposure in low incidence settings. Conversely sensitivity results were similar to those recently reported in the literature because based on patients with active disease [5], [16], [30].

In terms of parameters used to evaluate the accuracy of diagnostic tests it is important to consider that while sensitivity and specificity are easy and straightforward measures, they are limited and must be considered as surrogates for patient-important outcomes. There is still lack of adequate data on important outcomes such as accuracy of diagnostic algorithms (rather than single tests), incremental or added value of IGRAs, impact of IGRAs on clinical decision-making and therapeutic choices, and the prognostic ability of IGRAs to accurately identify individuals with LTBI who are at the highest risk for progressing to active tuberculosis and therefore are most likely to benefit from preventive therapy. These issues need to be evaluated for further studies.

New tools for a rapid diagnosis of active tuberculosis are needed especially for the smear negative and extra-pulmonary cases. In the smear negative tuberculosis group, the overall response to RD1 selected peptides was 64.3% and, among the tests used for comparison, a significantly higher sensitivity was found only for the QuantiFERON-TB GOLD In-Tube. Conversely the sensitivity for extra-pulmonary tuberculosis was 71.4% for the RD1 selected peptides and this value did not significantly differ from the results obtained by the other tests, with the exception of TST. However, in general, the size of these sub-groups of patients was small and no definitive conclusion can be drawn.

The rate of indeterminate results in our study was similar to the 3% to 4% rates observed in other studies [31]–[33]. Moreover, the proportion of patients with tuberculosis was no higher among those with indeterminate results. As expected, false-negative results to any of the immune assays considered were associated with factors known to cause anergy such as disseminated disease.

Taken together, our results suggest that none of the tests considered is accurate enough to be used in clinical practice to diagnose active tuberculosis, and new approaches should be considered. Recently, it has been shown that the discrimination of active tuberculosis from LTBI may be ameliorated by documenting recruitment of *M. tuberculosis*-specific lymphocytes to the site of the infection by RD1-specific ELISPOT assays [34]–[36] which may open a new strategy for the distinction of the two different status of tuberculosis.

Another recent study suggests that the combination of different immunodiagnostic tests may improve their diagnostic accuracy [21]. In fact it has been shown that, T-SPOT.*TB* or a new ELISPOT assay incorporating the Rv3879c to RD1 antigens, when used in combination with TST have an increased positive and negative likelihood ratio compared with single tests suggesting that this approach can be used to exclude active tuberculosis in patients with moderate to high pre-test probability of disease [21]. The results of the present study confirm and extend these findings. In fact, we confirmed the diagnostic performance of the commercial version of T-SPOT.*TB* when combined to TST, and additionally, we substantiated the data analyzing the results obtained by the assay based on RD1 selected peptides and the QuantiFERON-TB GOLD In-Tube. The likelihood ratio of a negative test result that was 0.38 with the assay based on RD1 selected peptides, became 0.19 when combined with a negative TST. Similarly, the combination of a negative result of the QuantiFERON-TB GOLD In-Tube assay with a negative TST scoring reached the lowest negative likelihood ratio of 0.07. This means that while a negative result to RD1 selected peptides test reduces 2.6-fold the odds of tuberculosis, a negative result to both tests, RD1 selected peptides and TST, would reduce the odds of tuberculosis 5.3-fold and using the combination of TST and QuantiFERON-TB GOLD In-Tube by 14.2-fold which may currently be the best available option to rapidly exclude tuberculosis by immunodiagnostic tests. Conversely, a positive result of the assay based on RD1 selected peptides with a corresponding likelihood ratio of 2.48 is of limited value and it is not significantly modified in those with positive results from either the assays based on RD1 selected peptides or the TST or QuantiFERON-TB GOLD In-Tube (likelihood ratio goes up to 2.92 and 3.21 respectively). Similarly, results on other tests combinations improve the positive likelihood ratio of the single test per se, but do not increase significantly that obtained by the combination of RD1 selected peptides with TST or QuantiFERON-TB GOLD In-Tube.

The higher sensitivity of combined use of the novel assay or commercial IGRAs with TST reflects the fact that patients who had a false-negative result with one test were distinct from those who had a false-negative result with the other. This implies that distinct immunologic processes underlie failure of these different, yet complementary, immune-based tests.

The study has some limitations. Not all individuals were tested by all the assays in parallel, as not all techniques for the different tests were established in the participating centers. In addition, the restricted number of immunocompromised patients does not allow a generalization of the results to this patients group. However, the prospective and multicenter design of the study, the high consistency of data across the different centers and the large number of patients enrolled to evaluate the diagnostic accuracy of different immune based tests in clinical practice render the results robust.

In conclusion, current approaches to elicit *M. tuberculosis*-specific immune responses in PBMC or in the skin by using either a broad or narrow spectrum of epitopes of RD1 mycobacterial antigens have a limited value for the diagnosis of active tuberculosis, as these tests do not reliably distinguish patients with active tuberculosis from those without. This is important to be considered in populations with a high pre-test probability of *M. tuberculosis* infection. However, the combined use of negative test results obtained by IGRAs or the test based on RD1 selected peptides with TST may enable rapid exclusion of tuberculosis.
