# Correction: Accuracy of Immunodiagnostic Tests for Active Tuberculosis Using Single and Combined Results: A Multicenter TBNET-Study

**DOI:** 10.1371/annotation/9c073390-ed15-44c9-bb85-b64e094e351a

**Published:** 2009-06-02

**Authors:** Delia Goletti, Stefania Carrara, Ornella Butera, Massimo Amicosante, Martin Ernst, Ilaria Sauzullo, Vincenzo Vullo, Daniela Cirillo, Emanuele Borroni, Roumiana Markova, Roumiana Drenska, José Dominguez, Irene Latorre, Claudio Angeletti, Assunta Navarra, Nicola Petrosillo, Francesco Nicola Lauria, Giuseppe Ippolito, Giovanni Battista Migliori, Christoph Lange, Enrico Girardi

The second author's name is incorrect. The correct name is: Stefania Carrara. The correct citation is: Goletti D, Carrara S, Butera O, Amicosante M, Ernst M, et al. (2008) Accuracy of Immunodiagnostic Tests for Active Tuberculosis Using Single and Combined Results: A Multicenter TBNET-Study. PLoS ONE 3(10): e3417. doi:10.1371/journal.pone.0003417

